# The effectiveness and safety of traditional Chinese herbal medicine for the treatment of male infertility associated with sperm DNA fragmentation: A protocol for systematic review and meta-analysis

**DOI:** 10.1097/MD.0000000000024918

**Published:** 2021-03-05

**Authors:** Qingrui Li, Chao Zhang, Chenxi Li, Xuyao Lin, Mingkai Wang, Lin Wu, Hua Li, Pule Ye, Guozheng Qin

**Affiliations:** a Beijing University of Chinese Medicine, Beijing; b Yunnan University of Traditional Chinese Medicine; c Hospital of Yunnan University of Traditional Chinese Medicine, Kunming, Yunnan, China.

**Keywords:** male infertility, protocol, sperm DNA fragmentation, systematic review, traditional Chinese medicine

## Abstract

**Background:**

Sperm DNA fragmentation (SDF) may hinder embryonic development and growth, increasing the risk of spontaneous miscarriage, and is considered an important factor affecting male infertility (MI). Traditional Chinese herbal medicine is considered effective in the treatment of MI due to SDF by nourishing kidney essence or promoting blood circulation for removing blood stasis. The objective of this systematic review protocol is to evaluate the effectiveness and safety of traditional Chinese herbal medicine on the treatment of MI associated with SDF.

**Methods:**

We searched the PubMed, Embase, Web of Science, Cochrane Library, CNKI, VIP Chinese Science, Technology Journal Database, and Wanfang Database until the end of 2020 for English and Chinese published literature. Randomized controlled trials (RCTs) to evaluate the effectiveness and safety of traditional Chinese herbal medicine for the treatment of MI associated with SDF will be included. Study selection and data extraction were performed independently by 2 reviewers, and the quality evaluation and risk assessment were assessed by the Cochrane collaboration's tool, and use the RevMan 5.3 software for meta-analysis.

**Conclusion:**

This study will evaluate the efficacy and safety of traditional Chinese herbal medicine for the treatment of MI due to SDF, which may provide some help for the clinician's decision.

**Prospero registration number:**

CRD42020221053.

## 1 Introduction

Infertility is a disease characterized by the failure to achieve a successful pregnancy after at least of 12 months of regular and unprotected sexual intercourse.^[1]^ The incidence of infertility ranges from 10% to 20% of reproductive-aged couples in the world.^[2,3]^ The success of a pregnancy is influenced by both men and women, and male factor of infertility reportedly accounts for nearly 50% of all infertility cases.^[4]^

Male infertility (MI) is a complex multifactorial disease, and there are many reasons for MI, such as congenital or genetic abnormalities, urogenital tract infections, varicocele, endocrine disturbances, malignancies, and immunological factors.^[5]^ Currently, accumulating evidence suggests that sperm DNA fragmentation (SDF) is one of the main factors affecting MI,^[6,7]^ and is associated with reduced pregnancy rates, fertilization rates, and embryo quality, and increased miscarriage rates.^[8]^ Studies have found that abnormal packaging and segregation of chromatin, abnormal cell apoptosis, oxidative stress, endogenous caspases and endonucleases, and exogenous factors may all affect the SDF.^[9–11]^ Some assisted reproductive technologies (ART), including in vitro fertilization and intracytoplasmic sperm injection have been applied to treat MI. The couple might be counseled to pursue ART when SDF is higher than 30%. However, the use of these techniques may increase the risk of birth defects in the offspring. Additionally, some drugs for MI have obvious side effects. Therefore, seeking effective natural remedies to treat MI is still the principal alternative. Traditional Chinese medicine (TCM) has been employed to treat MI for >2000 years, which can be caused by multiple TCM syndromes.^[12]^ The TCM therapy for MI has effects on multiple targets, pathways, and systems to improve pregnancy rate and sperm parameters. Traditional Chinese herbal medicine is a major modality based on TCM theory that prevent oxidative stress, modulate the proliferation and apoptosis of germ cells, supply trace elements, improve semen quality and the pregnancy rate, and alleviate inflammation in both natural conception and with the use of ART.^[13,14]^ Moreover, growing evidence suggests that traditional Chinese herbal medicine also prove helpful in preventing SDF by having a positive effect on oxidation, hormonal balance, and immune system.^[15,16]^ However, there is no systematic review and meta-analysis of traditional Chinese herbal medicine treatment MI due to SDF. The review aims to evaluate the effectiveness and safety of traditional Chinese herbal medicine as a clinical complementary treatment for MI associated with SDF.

## 2 Methods

### 2.1 Study registration

This protocol has been registered at PROSPERO (registration number: CRD42020221053). This protocol refers to the statement of Preferred Reporting Items for Systematic Review and Meta-analysis Protocols (PRISMA-P). And we will report the systematic review by following the PRISMA statement.

### 2.2 Inclusion criteria

#### 2.2.1 Type of studies

In this study only randomized controlled trials (RCTs) will be included. Other studies such as observational studies, retrospective analyses, self-controlled trials, patient series, case reports, reviews, animal studies, and laboratory in vitro studies will be excluded.

#### 2.2.2 Participants

##### 2.2.2.1 Included population

Men (aged 18–60 years) who have been diagnosed as MI associated with SDF according to the World Health Organization guidelines (WHO, 2010)^[17]^ will be included in this review.

##### 2.2.2.2 Excluded population

Men proven to have a living child, infertility couples with abnormalities only in the female factor will be excluded in this review.

#### 2.2.3 Interventions

We will only include studies which interventions involved traditional Chinese herbal medicine treatments.

#### 2.2.4 Outcomes

##### 2.2.4.1 The primary outcome

The primary outcome is SDF index that measured by sperm chromatin structure assay, terminal deoxyuridine nick end-labeling assay, comet assay, sperm chromatin dispersion assay, acridine orange test.

##### 2.2.4.2 The secondary outcome

The secondary outcomes of the review will include as followed: sperm concentration, progressive motility sperm, sperm morphology.

### 2.3 Search strategy

We will perform a search of the following databases from start to December 2020: PubMed, Embase, Web of Science, Cochrane Library, CNKI, VIP Chinese Science, Technology Journal Database and Wanfang Database. Search terms include: “MI,” “Male subfertility,” “Sperm,” “DNA fragmentation” and “TCM,” “Chinese medicine,” “Chinese herbal medicine,” “Proprietary Chinese medicine” and “RCTs,” “controlled clinical trial,” “randomized,” “controlled,” “trial,” “random”. Chinese search will use the Chinese form of the above terms. The search strategy for PubMed is shown in Table 1.

**Table 1:** PubMed search strategy.

Number	Search terms
1	MI
2	Male subfertility
3	Sperm
4	DNA fragmentation
5	1 OR 2 OR 3 OR 4
6	TCM
7	Chinese medicine
8	Chinese herbal medicine
9	Proprietary Chinese medicine
10	6 OR 7 OR 8 OR 9
11	RCTs
12	Controlled clinical trial
13	Randomized
14	Controlled
15	Trial
16	Random
17	11 OR 12 OR 13 OR 14 OR 15 OR 16
18	5 AND 10 AND 17

### 2.4 Study selection and data extraction

**Figure 1:**
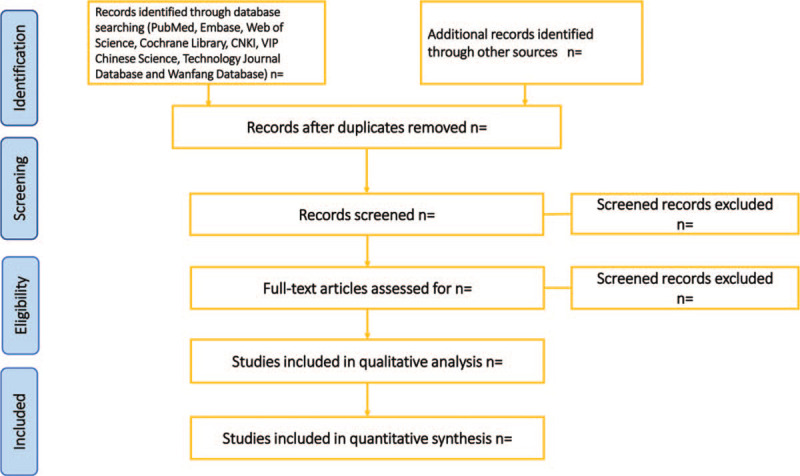
PRISMA flow diagram. PRISMA = Preferred Reporting Items for Systematic reviews and Meta-Analyses.

Two investigators (QRL and CZ) will independently filtrate the title and abstract of each record to exclude articles do not meet the study using Endnote X9 software. The full text of the qualified literature will be further investigated based on the inclusion criteria. All disagreements between the 2 investigators will be resolved after discussion with the third investigator (CXL). The flow chart of the selection process was summarized in Fig. 1. Two investigators (QRL and CZ) will extract data from the included trials independently and enter the data into a standard extraction form, which includes the following detailed information: general information (author, country, publication year), characteristics of participants, intervention method of control group and intervention group, and outcomes. If the data are incomplete, we will contact the author to obtain complete data. A third investigator (CXL) will be consulted for an expert opinion in the event of disagreements between the 2 investigators.

### 2.5 Risk of bias assessment

Two investigators (QRL and CZ) will independently assess the risk of bias in accordance with the Cochrane risk of bias tool^[18]^: random sequence generation, allocation concealment, blinding of participants and personnel, blinding of outcome assessment, incomplete outcome data, selective reporting, and other bias. The evaluation results are expressed as low risk, unclear. Any disagreements between the 2 investigators will be resolved after discussion with the third investigator (CXL).

### 2.6 Data analysis and synthesis

All research data will analyze use RevMan 5.3 software. The included literature will be tested for heterogeneity. When *P* ≥ .1, *I*^2^ < 50%, multiple similar studies are homogeneous, and a fixed-effects model is required for data analysis; when *P* < .1, *I*^2^ ≥ 50%, multiple similar studies are heterogeneous, and the random effects model needs to be used for progressive data analysis. For continuous variables, the weighted mean square error is selected when the measurement tools are the same, and the standardized mean square error is selected when the measurement tools are different. The interval estimation can be expressed with a 95% confidence interval.

### 2.7 Subgroup analysis

Subgroup analysis will be performed according to age, ethnicity, and duration of intervention.

### 2.8 Sensitivity analysis

Sensitivity analysis will be used to detect the sources of heterogeneity between the studies, and compare the results of the meta-analysis by comparing all the included literature and excluding the literature that caused the heterogeneity.

### 2.9 Publication bias

When included literature >10 trials, using the funnel plot to test whether there is publication bias.

### 2.10 Ethical review

Since our research did not have close or direct contact with every patient, the issue of ethical review does not exist.

## 3 Discussion

The integrity of sperm DNA is critical to the fertilization and embryonic development, and SDF can result in damaged sperm and lead to infertility. Several studies have shown that infertile men show higher SDF levels compared with fertile men, the fertilization rate is close to zero when SDF is >30%,^[19]^ high levels of SDF can cause recurrent miscarriage and affect the success rate of ART,^[20,21]^ which suggests that SDF could be evaluated as a new marker in the semen analyses for MI workup and a useful tool for MI diagnosis and management.

In China, TCM has a long history and great deal of experience in the treatment of MI by boosting the function of Sertoli cells and Leydig cells, regulating the hypothalamic-pituitary-testicular axis, preventing oxidative stress, modulating the proliferation and apoptosis of germ cells, ameliorating the microcirculation of the testis, and alleviation of inflammation.^[22]^ The TCM therapy for MI mainly by regulating the testis and improving the condition of the body having few side effects, instead of complementing hormones. Traditional Chinese herbal medicine is a major modality based on TCM theory that treats reproductive imbalances both for men and women. Herbal formulas, some of which increase circulation, reduce oxidation, and inflammation, may help to improve male factor fertility and prevent SDF,^[15,16]^ but its efficacy and safety for the treatment of MI associated with SDF have not been systematically evaluated. Therefore, we will use systematic review and meta-analysis to evaluate the effectiveness and safety of traditional Chinese herbal medicine for the treatment of MI associated with SDF, expecting that the review could provide new suggestions for future researches on TCM treatment for MI.

## Author contributions

**Data curation:** Qingrui Li, Chenxi Li, Chao Zhang.

**Formal analysis:** Xuyao Lin, Mingkai Wang, Lin Wu.

**Methodology:** Qingrui Li, Chenxi Li, Chao Zhang.

**Project administration:** Guozheng Qin.

**Software:** Hua Li, Pule Ye.

**Supervision:** Chao Zhang, Guozheng Qin.

**Validation:** Chao Zhang, Guozheng Qin.

**Writing – original draft:** Qingrui Li, Chenxi Li.

**Writing – review & editing:** Chao Zhang, Guozheng Qin.
